# Finishing Analysis of Dental Outcome (FADO) as a New Guide in Orthodontic Treatment

**DOI:** 10.7759/cureus.34808

**Published:** 2023-02-09

**Authors:** Oscar Zapata, Diana Barbosa, Alvaro Carvajal, Carlos M Ardila

**Affiliations:** 1 Basic Sciences, Universidad de Antioquia, Medellin, COL

**Keywords:** aesthetic dentistry, clinical practice guideline, dental finishing, orthodontic treatment dental esthetics, orthodontics

## Abstract

Objectives: Orthodontic treatment has both functional and esthetic goals. The finishing phase is one of the most important and seeks to solve occlusal, dental, functional, and esthetic problems, offering the possibility of obtaining the best possible outcome. This paper aims to describe the Finishing Analysis of Dental Outcome (FADO) guide as a tool to improve orthodontic treatment results.

Methods: A literature review of finishing procedures and orthodontic treatment outcomes was made. Moreover, current parameters of the esthetics of the smile were considered. The information was systematized to produce a protocol applicable to practice. This guide includes the assessment of mini-esthetic, micro-esthetic, and occlusal results using the Cast Radiographic Evaluation of the American Board of Orthodontics. Treatment options for each item were also suggested. Clinical cases were used to illustrate the application of the guide.

Results: The FADO Guide is presented and explained to give the clinician a systematic and orderly guide to achieve the best possible results in orthodontic treatment. To fulfill the FADO Guide, cast models, a panoramic radiograph, smile photographs (frontal and lateral), and an intraoral frontal photo are needed. The FADO Guide includes four sections: mini-esthetic evaluation (the evaluation of the smile), micro-esthetic evaluation, occlusal analysis, and radiographic analysis.

Conclusion: The FADO guide presents a systematic approach to the application of a finishing protocol in orthodontics. Furthermore, this guideline can improve clinical practice and be used as a checklist to optimize clinical outcomes.

## Introduction

The management of craniofacial and occlusal alterations begins with a complete clinical evaluation and a correct diagnosis. This provides the basis for establishing the treatment objectives to be fulfilled as the orthodontic treatment progresses. In this sense, the finishing stage begins at the moment the diagnosis is made and the treatment is planned [1].

Conventional orthodontic treatment includes three phases: (1) alignment and leveling, (2) closing of spaces, and (3) finishing and details [1,2]. In the finishing phase, dental alterations should be corrected in detail, including torque, tip, and occlusal relationship as well as mini and micro esthetic problems, including periodontal and dental morphology details [1].

The occlusal and radiographic characteristics of finished patients have been evaluated using the Objective Grading System (OGS), now named Cast Radiographic Evaluation (CRE), established by the American Board of Orthodontics (ABO) [2,3]. Regarding finishing protocols, different studies have shown that the use of finishing protocol improved occlusal outcomes and reduced the CRE score [4-6]; however, it was found satisfactory scores for only 50% of the population of finished patients [7,8]. These results motivated the search for a finishing protocol that could be used to guide the clinician in the improvement of clinical outcomes. In addition to the occlusal aspects evaluated by the CRE guide, the clinician must consider other occlusal and esthetic characteristics in the finishing phase of orthodontic treatment. Various studies have shown that the perception of orthodontic treatment results between patients and orthodontists is different [9]. While patients consult for esthetic reasons and the perception of the treatment results is concentrated on this factor, on the other hand, orthodontists evaluate the esthetic, occlusal, and functional factors, among others [9]. To our knowledge, the protocols used for the evaluation of the outcome of orthodontic treatments do not contemplate esthetic parameters such as the midline, smile arc, smile line, embrasures, connectors, line smile, and midline, among others [2,3,10]. These aspects are important in the perception of esthetics by orthodontists and laypeople [9,11]. Therefore, the current article presents the Finishing Analysis of Dental Outcome (FADO) guide, a systematic guide for finishing orthodontic treatments, proposed for guiding the clinician for achieving better occlusal and esthetic orthodontic treatment outcomes.

## Materials and methods

Search strategy

For the elaboration of this guide, a focused literature search was conducted using PubMed and LILACS databases. Google Scholar was also used. Keywords such as "orthodontic treatment outcome", “esthetics”, “smile”, “objective grading system”, “laypeople perception”, and “orthodontist perception” were used. Then, a process was implemented to research databases, utilizing Boolean operators (AND, OR): "orthodontic treatment outcome" AND “esthetics” AND “smile” OR “objective grading system” AND “laypeople perception” OR “orthodontist perception.”

Selection criteria

All types of studies published in English and Spanish were screened without date restriction. Hand search included a search of the Journal of Clinical Orthodontics, American Journal of Orthodontics and Dentofacial Orthopedics, Angle Orthodontics, Journal Dental Press Orthodontics, and European Journal of Orthodontics. Systematic reviews, randomized clinical trials, controlled clinical trials, case series, observational prospective, and retrospective studies, and narrative reviews which evaluate the quality of orthodontic treatments and the occlusal and esthetic factors related, were considered eligible for inclusion in this review. Observational studies that included children, dentofacial orthopedic treatments, and studies performed in patients with syndromes were excluded from the review.

Data extraction

The information included in the selected papers was extracted independently and in duplicate by two review authors (A.C. and O.Z.). Disagreements were solved by a discussion with a third evaluator (D.B.). The primary outcome was orthodontic treatment evaluation. The secondary outcome included tools for grading the quality of orthodontic treatment and other aspects for improving the orthodontic treatment results.

All relevant articles that contribute aspects aimed at improving orthodontic treatments were included in the preparation of the FADO Guide. The guide was developed by the authors based on existing literature on the subject and as part of an institutional process to improve the results of orthodontic treatments. The guide was presented to a group of professors and postgraduate students who, in different meetings, made suggestions for improving the guide and began its application in the postgraduate clinics as part of a complete protocol. The criteria included in the guide were those that the literature refers to as the most detected in the smile by dentists, orthodontists, and laypeople. The occlusal and radiographic criteria were described by the American Board of Orthodontics in the Cast radiographic evaluation. For the evaluation of patients using the FADO Guide, intraoral and smile photographs (frontal and lateral), cast models, and good-quality digital panoramic radiography are needed. It is important to note that silicone alginate impressions were taken from the upper and lower arches with the inclusion of the second molars; these impressions were cast in type 1 stone plaster. The models must be of good quality, without bubbles or casting defects in dental morphology. These clinical records should be taken at the end of the second phase, before the beginning of the orthodontic treatment finishing phase.

Three essential aspects must be present for the development of the FADO Guide: esthetic aspects, occlusal evaluation, and radiographic characteristics.

1. Esthetic aspects: One of the most relevant aspects in the evaluation of the results of orthodontic treatment is the smile [12,13]. The FADO Guide includes smile evaluation according to Sarver concepts. Sarver divided esthetic aspects into three factors: macro-esthetic, mini-esthetic, and micro-esthetic [13]. The macro-esthetic aspects must be solved during the previous phases of orthodontic treatment. The FADO Guide evaluates the most relevant factors of mini-esthetic and micro-esthetic using the frontal and lateral smile photographs. 1. Extraoral 1:1 photograph was taken using the ring flash. The following parameters were used: shutter speed 1/125, aperture 1/18, sharpness=1, contrast=-3, saturation=-2, and hue=-1.

**Figure 1:**
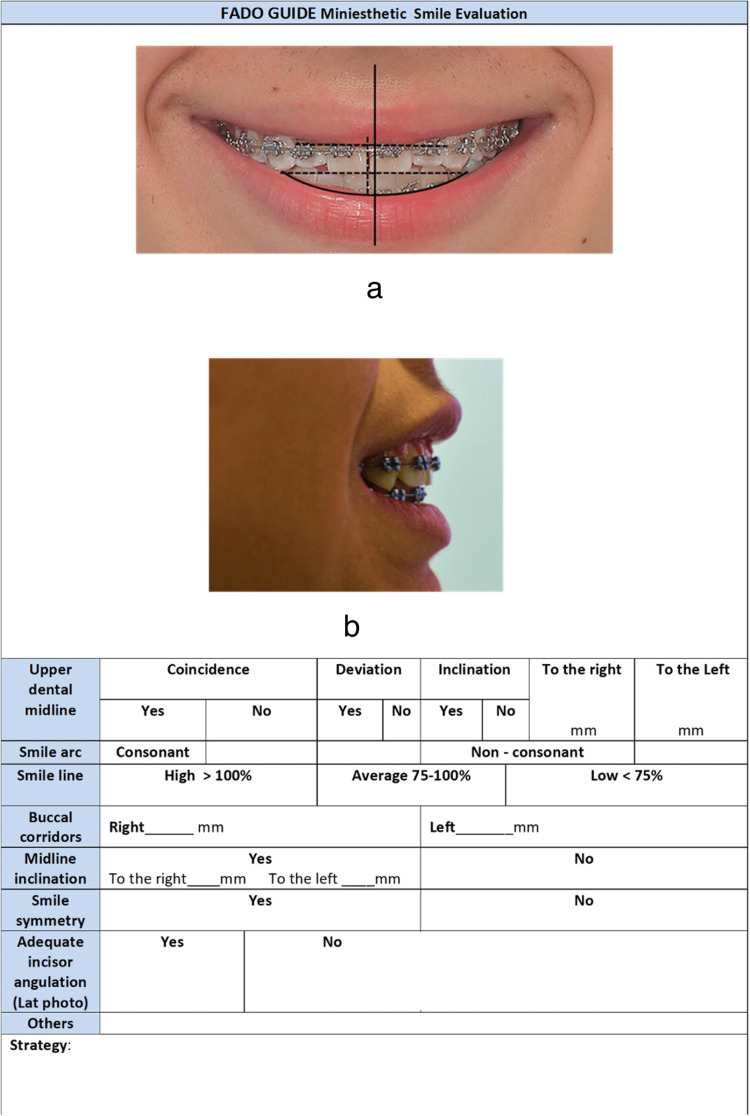
The items that should be included in the evaluation of the mini-esthetic smile evaluation. (a) Mini-esthetic (b) Incisor angulation evaluation

1.1 Mini-esthetic [13-15]: Figure 1 shows all the items that should be included in the evaluation of the frontal smile photography.

Upper dental midline deviation: The maxillary dental midline should be evaluated relative to the midsagittal plane. The reference line is drawn from the nasal tip to the middle of the philtra columns. The distance from this line to the contact between the upper central incisors should be zero. : The evaluation of the upper dental midline should be made with the lowest point of the labial philtrum as a reference point. However, adopting the lowest point of the labial philtrum as a reference point has some problems, because it is a soft tissue area that is subject to change with bruises, accidents, and deformities, in addition to cosmetic procedures such as fillers. Ideally, they should coincide, although some studies show that minor deviations are not perceived by laypeople [15]. It is important to mention that alterations in tooth size can produce midline deviation; therefore, these must be corrected at the beginning of the treatment by adding or subtracting dental material. Other midline alterations should be corrected with asymmetric extractions, stripping, or distal movement on the side opposite the deviation, as well as the use of medium-line elastics as early as possible. Figure 1a shows a right upper and lower midline deviation of 1 mm. Any discrepancy must be recorded in mm as a midline deviation.

Smile arc: It is defined as the relationship of the curvature of the incisal edges of the maxillary incisors and canines to the curvature of the lower lip in the posed smile. This relationship should be coincidental. In an esthetic smile, the incisal edges of the maxillary anterior teeth should follow a somewhat convex course that coincides with the curvature of the lower lip [16]. In the ideal smile, there must be a concordance between these two lines, without exposure of the lower incisors [17]. In some cases, the extrusion of the upper central incisors or a slight intrusion of the lateral ones is required to achieve the concordance of the arc. The use of vertical elastics can help as can the use of a thinner wire in the upper arch than in the lower one. Gender differences should be considered when evaluating and treating the smile arc [17]. These gender differences refer to the fact that women with a posed smile expose more amount of dental structure than men; moreover, women have a greater amount of gingival exposure in a posed smile than men. Likewise, women on average have a lower labial thickness in relation to men [17]. Figure 1a describes a non-consonant smile arc. Any discrepancy must be recorded using an X on the respective column.

Smile line: Although this factor is influenced by the age and gender of the patient [18], gum exposure when smiling ideally does not exceed 2 mm in young adult patients [12,13,19]. It could be high (more than 100% of the crown exposure of the upper central incisor), average (between 75% and 100% of the central incisor exposure), or low (less than 75% of the central incisor exposure). However, women tolerate a little more gum exposure than men when they smile [18]. If the cause of the problem is anatomical or functional (such as a short lip, a vertical excess of the maxilla, or a labial hyperfunction, etc.), a surgical procedure or Botox may be needed. On the other hand, if a dental or periodontal problem is the cause, intrusion movements of the anterior upper teeth or coronal lengthening surgical procedures could be used. Each case must be evaluated individually. Figure 1a presents a low smile line. A line is drawn on the lower edge of the upper lip and the percentage of exposed clinical crown is calculated, in a posed smile and is recorded in the corresponding box by marking with X.

Buccal corridors: The accepted amplitude for the buccal corridors varies from 8% to 22% of the total smile width, or from 5.0 mm to 16.0 mm, with an ideal value of 11.6 mm [19,20]. There is a preference for broad smiles with narrow and medium buccal corridors, which vary between 0% and 10% of the total amplitude of the smile. The presence of very narrow corridors can give the impression of a prosthetic smile [19,20]. Therefore, controlling the torque of the maxillary buccal segments and the expansion of the arches makes it possible to achieve or maintain the appropriate buccal corridors. The buccal corridors are determined by the distance between the internal surface of the labial commissure and the vestibular surface of the most posterior tooth exposed in a posed smile. This distance is recorded in mm, on the corresponding side.

Upper midline inclination/incisor angulation: The parallelism in the inclination of the maxillary dental midline, concerning the facial midline, may be even more important than the coincidence between them [15]. A midline inclination greater than 3 mm can be considered attractive, demonstrating that deviation from the midline can be acceptable if the area of dental contact is vertical [20]. The ideal would be to work on strategies for correcting the inclination of the midline before finishing orthodontic treatment. These strategies include repositioning braces, verticalization of incisors, mini-screws, or surgical management of skeletal discrepancies in cases where this aspect was not considered in the treatment planning. The inclination of the incisors is determined by observing the dental protrusion, its relationship with the occlusal plane and the overjet, as well as the position of the upper lip. It is influenced by the facial type and the width of the upper arch. It must be marked with an X in the corresponding column.

The symmetry of the smile: The position of the perioral soft tissues has a great influence on smile symmetry [19]. A ratio close to 1:1 between the left and right sides is considered harmonic. In cases of asymmetric smiles caused by differences in muscle tone, myofunctional therapy can help overcome this deficiency and restore the symmetry of the smile [16,20]. Likewise, aesthetic management of perioral tissues can be an effective adjuvant to improve treatment results. The similarity between the appearance of the smile between the right side and the left side, according to the facial midline, should be observed. It must be registered with an X, in the corresponding box.

Incisor angulation: The clinical expression of the upper and lower incisors’ relationship and their position concerning the lips should be evaluated. Torque management with rectangular wires and repositioning of brackets and elastics are used to correct these alterations [19]. Figure 1b depicts incisor angulation evaluation.

Other aspects: The appearance of the smile can be affected by different factors, including muscle hypermobility and facial asymmetry, among others [6,13,16]. All these aspects must be fulfilled in the register sheet to get the complete mini-esthetic evaluation (Figure 1).

**Figure 2:**
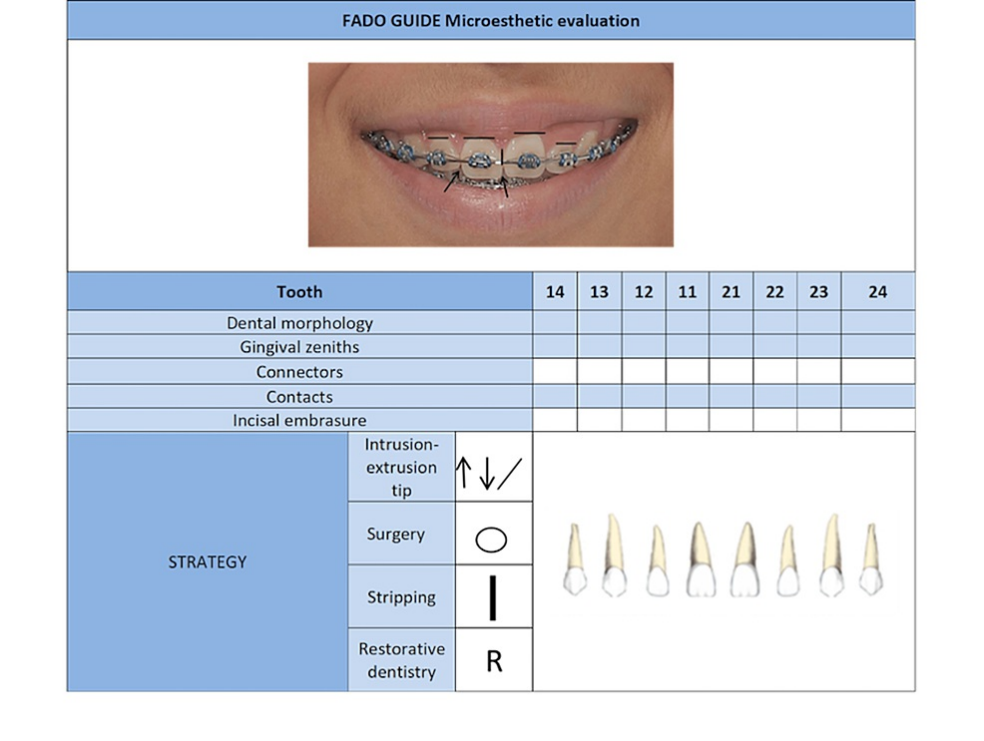
Different aspects that must be evaluated in micro-esthetic.

1.2. Micro-esthetic [14]: The details of micro-esthetic refer to the dental-periodontal relationship and anatomical alterations in tooth size and shape. Figure 2 shows the different aspects that must be evaluated, and the strategies for solving them.

Dental morphology: The length/width ratio of the tooth is an important aspect when evaluating micro-esthetic. Any alteration of this type should be corrected by adding or subtracting dental material as early as possible (e.g., Bolton discrepancies, macro, microdontia, etc.) [14].

Gingival zeniths: Ideally, the central incisors display a distal gingival zenith position deviation from the central axis, with a mean of 1 mm [21]. The zenith (or highest point of the gingiva in the vestibular) should be at the same height on the central and canine teeth and a little lower on the lateral ones [13,21]. This morphology is a factor that influences the perception of the result. Usually, the orthodontic treatment of zenith height alterations will include intrusion, extrusion, or inclination movements. If the origin of the problem is due to morphological alterations, it should be corrected as soon as possible by restorative dentistry or gingival surgery treatment [13]. Horizontal lines in Figure 2 depict different heights on gingival zeniths.

Connectors: The contact area between two teeth is called a connector. Depending on the tooth, it is a percentage of the length of the clinical crown. For the contact between the two centrals, it should be 50% of the clinical crown, for the contact between central and lateral it should be 40% and for the contact between lateral and canine, it should be 30% of the lateral [22]. Two simple strategies for solving this problem are to check teeth and/or to extend the contact point to the contact area through stripping. When taking this second option, it is important to consider the relationship between the width and the length of the crown [23] (See the vertical line in Figure 2).

Contacts: The contact zone between two teeth must be on a specific position of the interproximal area, normally located in the third incisal area. Morphological alterations can influence the position of the contacts. They have a high relationship with the connectors. A problem that can arise in this aspect is the appearance of black gingival space. Problems in this area should be corrected by evaluating the radicular inclination and the dental morphology by stripping [23].

Incisal embrasures: The triangular space incisal to the contact zone has an inverted V shape in the upper incisors. It must be symmetrical between the two centrals, slightly asymmetric between the central and lateral, and more marked between the distal of the lateral and the canine. These spaces are highly influenced by the length of the interdental connectors. Depending on their etiology, the alterations in incisal embrasures are corrected by changing the vertical positioning of the teeth or by adding composite [14]. They are shown in arrows in Figure 2.

**Figure 3:**
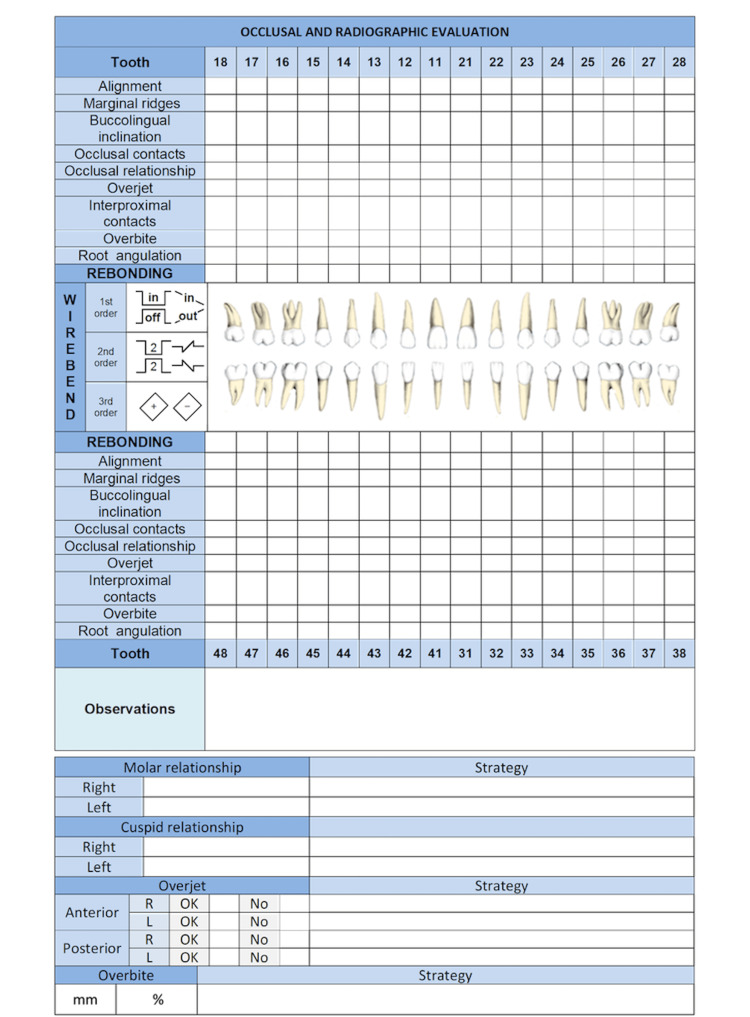
Occlusal and radiographic evaluation is illustrated.

2. Occlusal aspects: Occlusal aspects refer to the ideal characteristics of position, angulation, and height that the teeth should have when they are in occlusion. These are evaluated in cast models based on CRE created by the ABO [2]. Figure 3 shows a diagram that includes the different aspects contemplated by the CRE to be filled out by the clinician. The overbite criteria were added at the occlusal evaluation. Furthermore, the guide presents a section in which treatment strategy (bonding or repositioning), could be selected. The items that must be corrected are recorded in the observations section and then the strategies that the clinician will implement for their correction will be recorded. The aspects that must be included in the occlusal evaluation are described below:

Alignment: The palatal surface of the upper incisors, the vestibular surface of the lower incisors, and the occlusal grooves of the premolars and molars should be aligned. Depending on the prescription of braces used and the coronal morphology, ideally, the position of the braces should solve these problems. They can be solved by making first-order bends on the wire or by changing the position of the braces [24].

Marginal ridges: With the objective of the interdental bone ridges having the same height and thus promoting periodontal health, the difference in the vertical level of the marginal ridges of premolars and molars should not be greater than 0.5 mm. Sometimes intrusive/extrusive movement or tip bends must be made to correct them. Morphological alterations can affect the height of the ridges, so some authors suggest bonding the braces at the height of the marginal edges and not at a specific height according to the technique [25].

Buccolingual inclination or torque: To ensure adequate torque in the posterior sector, the upper vestibular cusps should be at a maximum of 1 mm apically concerning a horizontal plane. Likewise, in the lower arc, the lingual cusps should be at a maximum of 1 mm apically with respect to a horizontal plane [2,3]. The correction of this type of problem may require arc bends with torque corrections (individual, progressive, or continuous) in the wire. Another way to solve these problems would be to change the torque expression of the braces by selecting an appropriate prescription or by changing the position of the bracket by flipping it 180º [26].

Occlusal contacts: The occlusal surfaces of functional cusps of the upper and lower premolars and molars should contact [2,3]. The contacts in maximum intercuspidation should be monitored to give good occlusal stability. Repositioning the braces, the use of intermaxillary elastics, and thin and high-flexibility wires promote the obtaining of these contacts.

Occlusal relationship: The anteroposterior occlusal relationships should be from one tooth to two teeth or be in a range of 1 mm of that relationship [2,3]. When only upper or lower premolar extractions are performed, cases can be finished in Class II or Class III occlusal relationships. These objectives should ideally be met during the phase of closing spaces. In the finishing phase, the adjustment in these relations is achieved using class II or class III intermaxillary elastics, by interproximal stripping, bending the wire, or repositioning the braces.

Overjet: To avoid occlusal interferences during eccentric movements, the vestibular surfaces of the lower incisors and canines should contact the palatal surfaces of the upper incisors and canines in occlusion [2,3]. In the posterior sector, the vestibular cusps of the premolars and lower molars should contact the center of the occlusal surfaces of the premolars and upper molars. The use of class II, class III, or cross elastics and the management of the torque facilitate the achievement of this objective.

Interproximal contacts: The interproximal contacts are important to give dental stability and to avoid food packaging. Moreover, in the anterior sector, they have a high aesthetic impact. During the finishing phase, the consolidation of the arch must be ensured. To achieve this, elastic chains or continuous conjugated ligatures are used according to the individual case. The distal bend of the arch also prevents the reopening of any spaces [27].

Overbite: Adequate vertical overlap of the upper incisors over the lower incisors is important for the mandibular protrusive function. It should be between 20% and 30%. This aspect should ideally be corrected in the leveling phase. In some cases, intrusion overlays may be needed. This point is especially important in cases of gingival smiles [27]. Its correction may include the use of heavy arches, the use of anterior or posterior turbo bite, a change of the placement of the braces, or the use of bends on the archwire or mini-screws.

3. Radiographic aspects: Root inclination is one of the aspects to consider in the process of completing orthodontic treatment. For this, panoramic radiography is used as a diagnostic tool. The aspects that must be included in the radiographic evaluation are described below:

Root angulation: The roots of the maxillary and mandibular teeth must be parallel to each other and perpendicular to the occlusal plane [2,3]. Due to the difficulties involved in properly evaluating the canines using panoramic radiography, their inclination must be verified from the clinical point of view. Given the importance of mandibular excursion movements, root inclination must also be evaluated from the functional point of view. Tip alterations can be corrected by replacing braces or by making specific bends [26]. Figure 3 includes a row in which the clinician should record angulation problems that occur tooth by tooth.

## Results

The FADO Guide is a finishing checklist used to gather the completed information about the status of finishing cases. Each alteration should be corrected, and the solving strategy should be registered in the FADO Guide. To fulfill the FADO Guide, cast models, a panoramic radiograph, smile photographs (frontal and lateral), and an intraoral frontal photo are needed. The FADO Guide includes three sections: (1) esthetic evaluation that includes mini-esthetic and micro-esthetic evaluation (2) occlusal analysis and (3) radiographic analysis. Each of these parts is evaluated and recorded in its respective section. Each item has a section for recording the diagnosis or clinical problem and another section oriented to the clinical strategy for solving the problem. The tooth or problem area is marked with an X and the strategy for solving it is marked on the figure.

The FADO Guide should be filled in during the first appointment of the finishing stage. In the next appointments, the strategies for solving the problems should be applied. In the following appointments, the progress should be checked and colored in green when the process is completed. In the following section, the authors will demonstrate how to fill out the FADO Guide form:

Esthetic aspects

**Figure 4:**
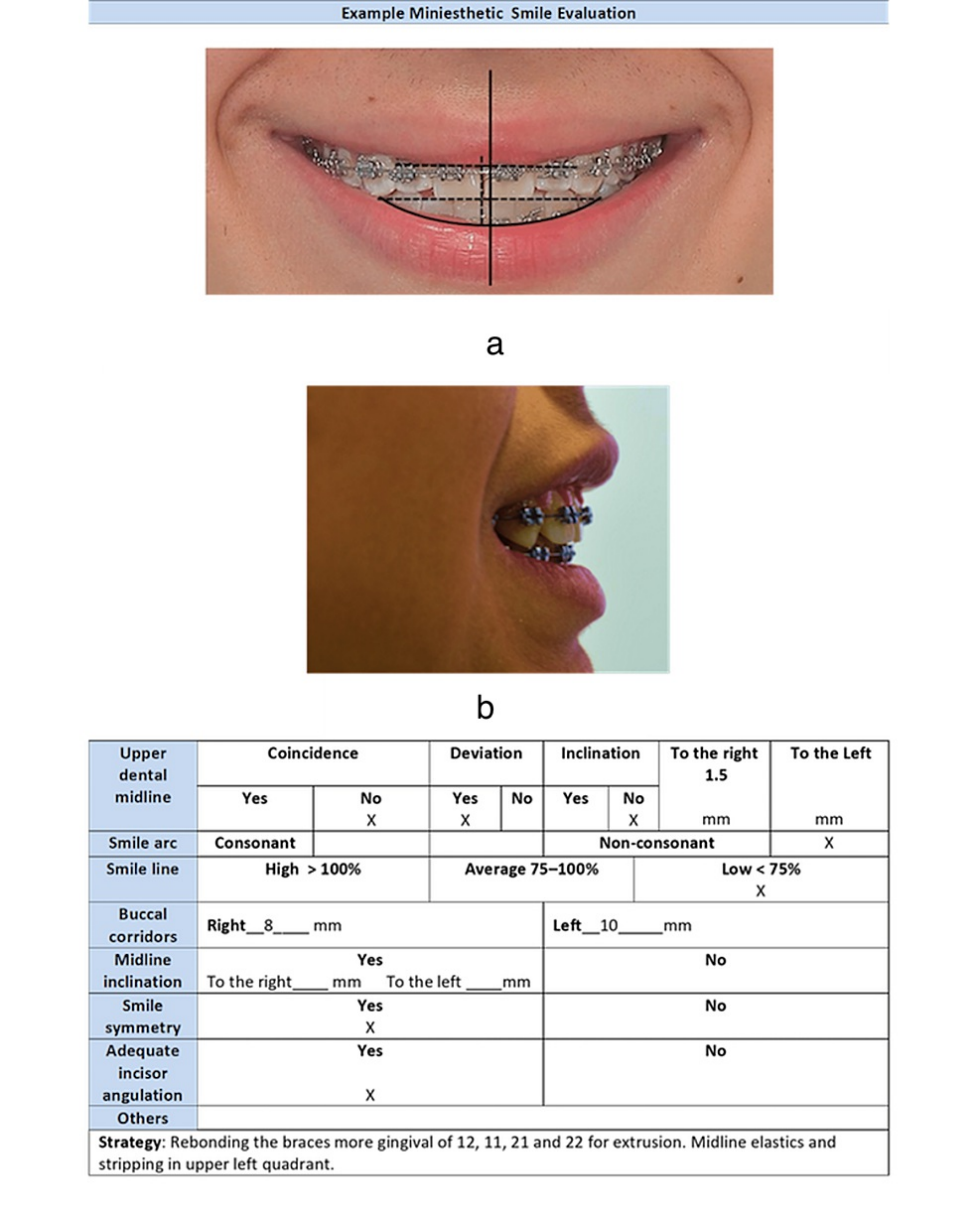
An example of a mini-esthetic evaluation is shown. (a) Frontal evaluation (b) Incisor angulation evaluation

Mini-esthetic: Figure 4 shows an example of how the FADO Guide should be filled out, registering the patient's mini-esthetic alterations in the corresponding box. Likewise, the selected treatment strategy is described. In this case, the upper midline has no coincidence with the facial midline and the smile arch is non-consonant. The smile line is low. In the strategy box, the clinician must write the strategy for solving the problem, in this case with extrusion of the upper incisor.

**Figure 5:**
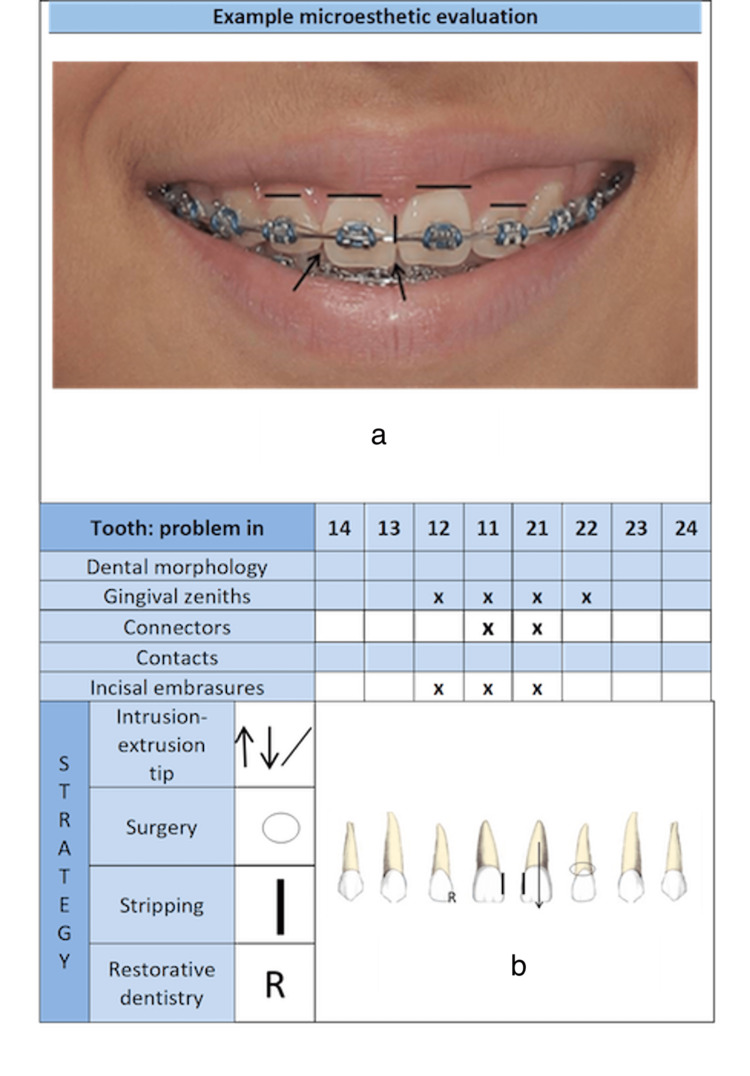
An example of a micro-esthetic evaluation is depicted. (a) Clinical evaluation (b) Micro-esthetic treatment

Micro-esthetic: Figure 5 presents the aspects that compromise the micro-esthetic of the patient, marked with a cross. Furthermore, the treatment strategy is recorded using the respective coding. In this section, the strategy for solving the problem must be drawn on the specific tooth, as in Figure 2. For example, the FADO Guide shows that 12 has problems with the morphology and needs restoration. Teeth 21 and 22 have problems in the zenith. The connector between 11 and 21 is high. Teeth 12, 11, and 21 have incisal embrasure problems. The problems in 21 will be solved by dental extrusion, so mark an arrow on this tooth. The stripping bar is sitting on the side where stripping is needed. Teeth 11 and 21 need some stripping so a black bar is marked on these teeth. The zenith problem in 22 will be solved by surgery so mark a circle over this tooth. Arrows are used for the direction of the movement; for dental and surgical procedures the respective symbols should be used (Figure 5).

Occlusal aspects

**Figure 6:**
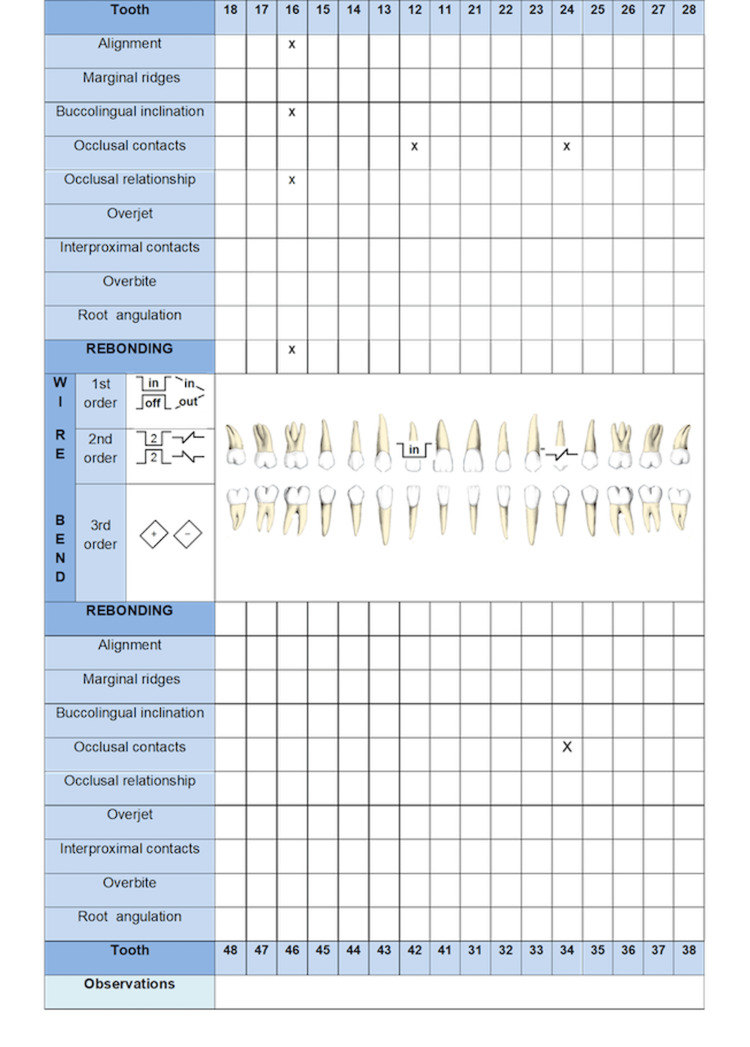
The occlusal alterations presented by the patient are presented.

Following the same method, the occlusal discrepancies must be filled in on this diagram. Figure 6 shows, marked with a cross, the occlusal alterations (according to CRE criteria) presented by the patient. Likewise, the treatment option defined for each tooth is presented. To present in a single figure the three planes of space, first-order bends are coded with the word IN or OUT, and those of the second order with the number 2 and included the shape of the bend (extrusive, intrusive, or mesial or distal radicular tip), according to the figure. An example is shown in Figure 6.

The FADO Guide example shown below has problems in 3 aspects (alignment, buccolingual inclination, and occlusal contacts), so in this case, we recommend the repositioning of the braces. Tooth 12 has alignment problems, which could be solved by bending the wire. Tooth 24 has an inclination problem that can be solved by second-order bending (see diagram). The color method could be used to follow the progress of the treatment (Figure 6).

For the evaluation of the molar and cuspid relationship, overjet and overbite, the clinician must fill in the FADO Guide and can write the selected strategy for solving the problem. In this case, the patient has problems in the left molar relationship, which should be solved by class II elastics. In overjet evaluation, the left lateral does not have adequate overjet; this would be solved by torquing the upper wire. There is no problem with overbites (Figure 6).

**Figure 7:**
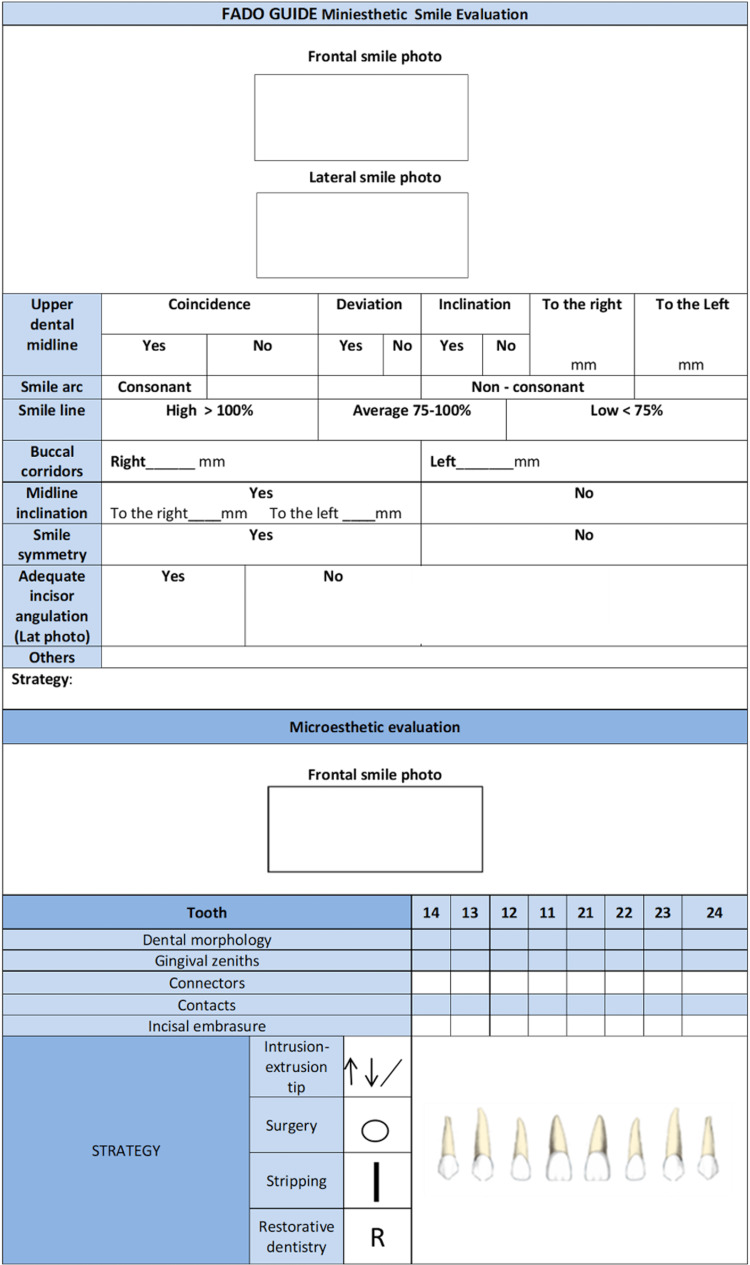
Mini-esthetic smile and micro-esthetic evaluation are displayed.

The FADO Guide helps the clinician follow a checklist for the finishing stage of the treatment. In this way, the clinician will promote excellence in orthodontic treatments. The FADO Guide is a useful tool for improving orthodontic results, and clinicians should seriously consider using it (Figure 7).

## Discussion

To our knowledge, the protocols used for the evaluation of the outcome of orthodontic treatments do not consider esthetic parameters such as the midline, the smile arc, the smile line, embrasures, connectors, line smile, and midline, among others [2,3,13]. These aspects are important in the perception of esthetics by orthodontists and laypeople [9,11] and have been incorporated into the FADO guide.

The FADO guide puts a lot of emphasis on the smile. Orthodontic treatment impacts the quality of life of people looking for better smiles [12,13,28]. Hence, orthodontic therapy should achieve a harmonious smile on the face [29]. Previously, orthodontists focused primarily on the occlusal and functional aspects of occlusion to resolve malocclusions. However, it has been observed that despite efforts to finish an ideal dental occlusion, the perception of the smile has not been the most appropriate [28]. A recent investigation observed that of the 68 smiling photos of finished cases evaluated for the American Board of Orthodontics examination, just two of the finished outcomes presented a perfect smile according to a panel of experts [30]. These results reframed the occlusal purposes of orthodontics and underlined the perceptive effect of the smile that should be achieved during therapy [28].

In addition to the occlusal aspects evaluated by the CRE guide, other occlusal and esthetic characteristics must be considered by the clinician in the finishing phase of orthodontic treatment [2,3]. Hence, the FADO guide contains relevant aspects such as the midline and overbite. It has been generally accepted that the two aspects are essential components of the smile [28].

On the other hand, the FADO guide does not include the esthetic macro aspects proposed by Sarver and Ackerman [13] because they are not part of the finishing phase. This aspect should be corrected during previous phases of the treatment [22]. Moreover, the FADO guide does not include the functional analysis either. These functional aspects must be evaluated using different parameters such as canine disocclusion, group contacts, and interferences on the balance side, among others, and should be evaluated by the clinicians before removing the appliances. Likewise, the relationship between the static results and the functional part could be an object of future research.

## Conclusions

The protocols used for the evaluation of the outcome of orthodontic treatments must consider esthetic parameters such as the midline, smile arc, smile line, embrasures, connectors, line smile, and midline, among others. These aspects are important for the perception of aesthetics by orthodontists and laypeople. The FADO guide presents a systematic approach to the application of a finishing protocol in orthodontics. Furthermore, this guideline can improve clinical practice and may be used as a checklist to optimize clinical outcomes.
